# The optimized drug delivery systems of treating cancer bone metastatic osteolysis with nanomaterials

**DOI:** 10.1080/10717544.2020.1856225

**Published:** 2020-12-18

**Authors:** Xi Cheng, Jinrong Wei, Qi Ge, Danlei Xing, Xuefeng Zhou, Yunzhu Qian, Guoqin Jiang

**Affiliations:** a Department of Surgery, The Second Affiliated Hospital of Soochow University, Suzhou, People’s Republic of China; b State Key Laboratory of Bioelectronics, Jiangsu Key Laboratory for Biomaterials and Devices, School of Biological Science and Medical Engineering, Southeast University, Nanjing, People’s Republic of China; c Center of Stomatology, The Second Affiliated Hospital of Soochow University, Suzhou, People’s Republic of China

**Keywords:** Osteolysis, cancer bone metastasis, drug delivery systems, nanomaterials

## Abstract

Some cancers such as human breast cancer, prostate cancer, and lung cancer easily metastasize to bone, leading to osteolysis and bone destruction accompanied by a complicated microenvironment. Systemic administration of bisphosphonates (BP) or denosumab is the routine therapy for osteolysis but with non-negligible side effects such as mandibular osteonecrosis and hypocalcemia. Thus, it is imperative to exploit optimized drug delivery systems, and some novel nanotechnology and nanomaterials have opened new horizons for scientists. Targeted and local drug delivery systems can optimize biodistribution depending on nanoparticles (NPs) or microspheres (MS) and implantable biomaterials with the controllable property. Drug delivery kinetics can be optimized by smart and sustained/local drug delivery systems for responsive delivery and sustained delivery. These delicately fabricated drug delivery systems with special matrix, structure, morphology, and modification can minimize unexpected toxicity caused by systemic delivery and achieve desired effects through integrating multiple drugs or multiple functions. This review summarized recent studies about optimized drug delivery systems for the treatment of cancer metastatic osteolysis, aimed at giving some inspiration in designing efficient multifunctional drug delivery systems.

## 1. Introduction

**Figure 1.**
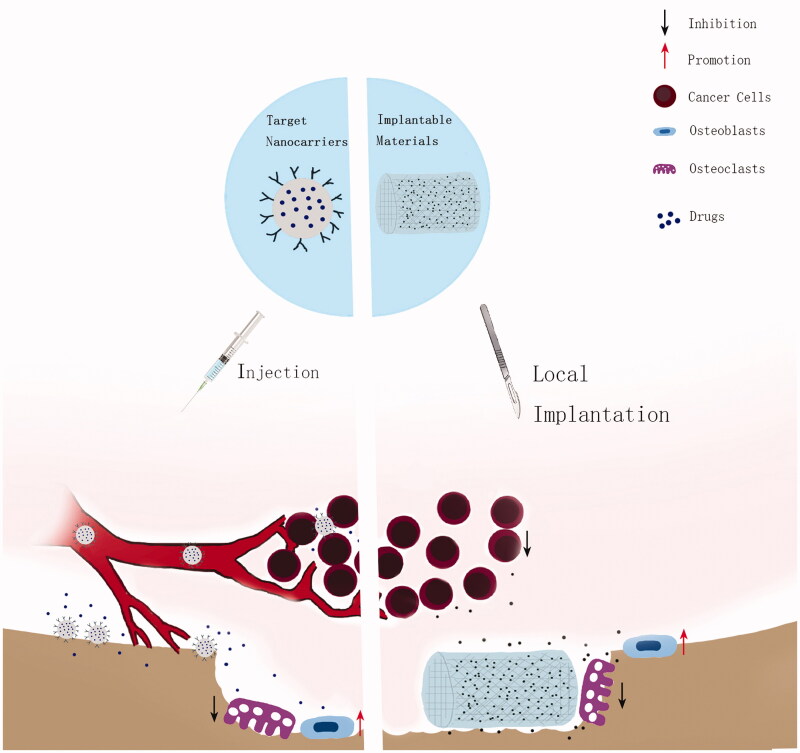
The graphical abstract of the work of optimized drug delivery systems.

Bone metastasis and concomitant complications are huge challenges for cancer treatment in the clinic. Bone metastases occur in about 70% of patients suffering from advanced breast cancer or prostate cancer and approximately 15–30% of patients with carcinoma of the lung, colon, stomach, bladder, uterus, rectum, thyroid, or kidney (Irelli et al., 2016; Sun, Han, et al., 2016). Escaping from the tumor *in-situ*, tumor cells enter circulation through blood vessels and lymph vessels and then travel all over the body to find fertile soil to proliferate (Croucher et al., 2016; Vinay & KusumDevi, 2016; Chen, Pei, et al., 2018; Ahangar et al., 2019). The special structure and microenvironment of the bone marrow are beneficial for tumor cells to grow (Ahangar et al., 2019). Tumor cells replicate in the new environment continually, develop into a metastatic tumor, and destroy the bone structure. Patients with bone metastasis suffer from bone pain, pathological fracture, hypercalcemia, spinal cord compression, and other skeletal-related events (Macedo et al., 2017; Turpin et al., 2020). Osteogenic metastasis or osteolytic metastasis both could happen. But osteolytic bone metastases account for approximately 80% of cancer bone metastases (Sun, Ge, et al., 2019). The ‘vicious circle’ occurring in the metastasized bone accelerates the cancer cells’ proliferation and bone destruction at the same time (Santini et al., 2010; Turpin et al., 2020). BP and denosumab are common medicine in the clinic but have many side effects. BP may cause mandibular osteonecrosis and impair renal function (Sun, Han, et al., 2016). Denosumab was reported to increase the risk of pancreatitis and endocarditis, erysipelas, and infectious arthritis (Jie et al., 2019). Some newly developed drugs have not been used clinically due to their hydrophobicity, short half-life, and other disadvantages (Chew & Danti, 2017). These undesired effects of systemic drug delivery arise from the uncontrollable ‘space’ and ‘time’ of drug reaction. Delightedly, nanotechnology and nanomaterials provide new strategies to build superior drug delivery systems that make drugs more efficient and secure to the body (Chen, Wang, et al., 2019). Many kinds of NPs were utilized as drug carriers and targeted destroyed bone areas to treat cancer and inhibit bone resorption, such as polymer NPs, liposomes, and silica NPs. Elaborate decorations with targeting ligands endowed particles the ability to target bone or special expression of cancer cells in the osteolytic microenvironment, optimizing the spatial distribution of drugs. The multifunctional implantable scaffolds or hydrogel materials can load a large number of drugs and release drugs locally in a more sustainable way, showing desired efficiency and minimal side effects and reducing the frequency of medication. In addition, smart drug release systems were designed to release drugs in response to some physical and chemical stimuli. Internal chemical characteristics of the osteolytic environment (low pH and high Ca^2+^ concentration) and external physical stimuli [near-infrared light (NIR), ultraviolet light, or ultrasound] both can be utilized as triggers of drug release and further optimize the timing of delivery. Figure 1 summarized the work of these optimized drug delivery systems.

## 2. Osteolytic cancer bone metastasis

**Figure 2.**
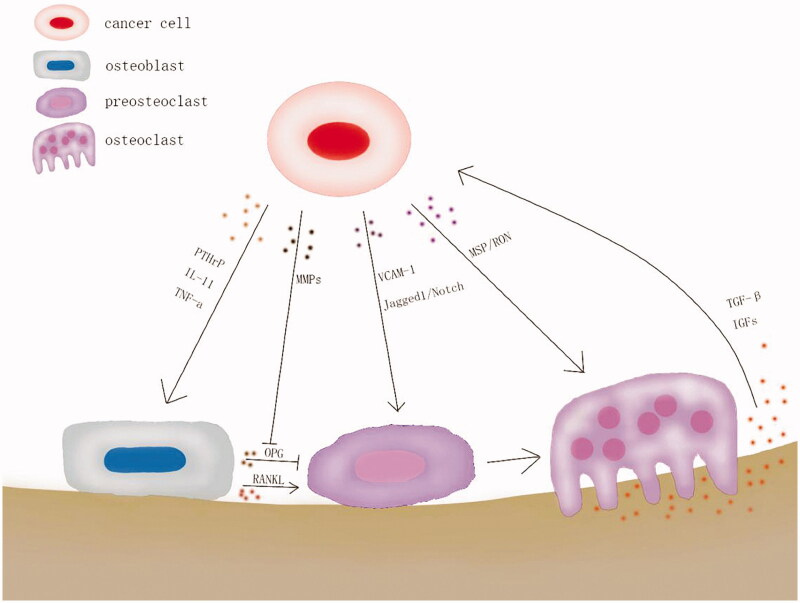
‘Vicious circle’ in osteolysis caused by cancer bone metastasis.

When metastatic tumor forms, tumor cells interact with normal cells in the vicinity, influence their cellular function, and destroy the primary microenvironment (Futakuchi et al., 2016; Shupp et al., 2018). Bone offers metastatic cells unique microenvironments termed ‘niches’ (Croucher et al., 2016; Ahangar et al., 2019). The slow blood flow in bone, mechanical property, various chemokines, and growth factors are beneficial to tumor cells’ growth (Zhu et al., 2018; Turpin et al., 2020). In the osteolytic microenvironment, the ‘vicious circle’ between cancer cells, osteoblasts, and osteoclasts facilitates the activation of osteoclasts and suppresses the action of osteoblasts. At the same time, cancer cells’ proliferation is also promoted continually (Futakuchi et al., 2016; Saracino et al., 2016; Turpin et al., 2020). In brief, metastatic cancer cells produce and excrete parathyroid hormone-related protein (PTHrP), IL-11, TNF-a, and other factors that act on the osteoblasts to promote the expression of receptor activator of nuclear factor-κB (NF-κB) ligand (RANKL). RANKL combines with receptor activator of NF-κB (RANK) on the membrane of preosteoclasts to accelerate osteoclast formation and maturity (Wu et al., 2020). Cancer cells also express the Notch ligand Jagged1 and VCAM-1 to promote the activation of preosteoclasts (Lu et al., 2011; Sethi et al., 2011). The bone matrix contains a variety of growth factors, such as insulin-like growth factors (IGFs), transforming growth factor β (TGF-β), fibroblast growth factors (FGFs), platelet-derived growth factors (PDGFs), and bone morphogenetic proteins (BMP) (Kagiya, 2015; Vinay & KusumDevi, 2016). These growth factors are released from the bone matrix during the bone resorption caused by mature osteoclasts. TGF-β acts on cancer cells to facilitate its proliferation and production of more PTHrP. Osteoprotegerin (OPG), also produced by osteoblasts, is a soluble RANKL decoy receptor that can prevent osteoclasts formation and osteoclastic bone resorption (Wu et al., 2020). However, the production of OPG is suppressed by matric meralloproteinases (MMPs) during this ‘vicious circle’ (Lu et al., 2009). Additionally, cancer cells secrete a macrophage-stimulating protein (MSP) to promote osteoclasts activation directly via the receptor on the osteoclasts, RON tyrosine kinase (Andrade et al., 2017). The diagram description of the interaction between cells in the osteolytic microenvironment is shown in Figure 2. Also, many microRNAs are involved in osteolysis, such as miR-21, miR-133a, miR-33a, miR-190, and miR-223 (Probert et al., 2019).

Nowadays, radiotherapy, surgery as well as systemic drug therapies (chemotherapy, hormone therapy), and targeted therapy are effective clinical therapies for osteolysis. The efficacy of BP appeared to be time-dependent and they were effective after 6–12 months of therapy (Irelli et al., 2016). Denosumab is a kind of human monoclonal antibody that combines especially with RANKL to inhibit the activation of osteoclasts. Many experiments showed that Denosumab was more effective than zoledronic acid (Zol or ZA) and caused fewer side effects (Stopeck et al., 2010; Irelli et al., 2016; Chen, He, et al., 2019; Turpin et al., 2020). However, Denosumab cannot accumulate in the bone like BP and its effect is reversible after treatment discontinuation (Macedo et al., 2017).

## 3. Therapeutic agents

### 3.1. Chemotherapeutic drugs

The theme of treating osteolysis is killing cancer cells and suppressing osteoclasts synchronously. So the optimized drug delivery systems usually loaded one kind of anticancer drugs like paclitaxel (PTX), doxorubicin (DOX), platinum (Pt), and one kind of anti-osteoclasts drugs simultaneously. Here, we emphatically introduced potent drugs and novel drugs for suppressing osteoclasts.

BP is a stable analog of pyrophosphate by replacing the P–O–P bonds with P–C–P bonds (Rudnick-Glick et al., 2016). This makes BP enzymatically stable. BP has a high affinity to the bone mineral hydroxyapatite (HA) and has been exploited as a targeting ligand. Osteoclasts can take up and be suppressed by BP chelating to bone (Zeng et al., 2019). In addition, BP has been revealed to be able to inhibit cancer cells (Robert & Reinhold, 2015; Santini et al., 2015). Hydroxyapatite nanoparticles (nHA) was always applied in bone repairing because of its good biocompatibility to the bone and fine adsorption to BP. Recently, nHA has been found to be able to inhibit several kinds of cancer cells through mitochondrial-dependent apoptosis and have special functions of lessening osteolysis caused by cancer metastasis (Zhang, Zhou, et al., 2019). The transcription factor Gli2 is a key regulator of PTHrP (Vanderburgh et al., 2019). And GANT58 is a Gli2 antagonist that inhibits Gli2 nuclear translocation and PTHrP expression in tumor cells to play a role in hindering the ‘vicious circle’ (Vanderburgh et al., 2019). Gallium (Ga) is a semi-metallic element with a chemical affinity for biological HA, anti-resorption activity and anticancer ability. Ga significantly decreased osteoclasts differentiation and activity without negative interaction with osteoblasts (Strazic-Geljic et al., 2016). Odanacatib and balicatib, inhibitors of cathepsin K, can suppress bone resorption (Saracino et al., 2016). Mature osteoclasts express cathepsin K which is a kind of lysosomal cysteine protease essential for collagen and elastin degradation during bone resorption. Table 1 collected some reported effective drugs for suppressing osteoclasts and some other potential drugs (Vinay & KusumDevi, 2016).

**Table 1.** The drugs with contributions to block ‘vicious circle.’

Drugs	Mechanism	Reference
BP	Non-nitrogen containing: inhibiting ATP hydrolysis Nitrogen containing: prevents protein prenylation of small GTPases	(Vinay & KusumDevi, 2016); (O’Carrigan et al., 2017); (Horne et al., 2018)
Denosumab	Antibody to RANKL	(Gul et al., 2016); (Lipton et al., 2016); (Stopeck et al., 2016)
Odanacatib and balicatib and L-235	Inhibit cathepskin K	(Saracino et al., 2016); (Duong et al., 2014)
BTZ	Inhibit NF-kB pathway in osteoclast	(Zhu et al., 2018); (Wang, Cai, et al., 2018)
GANT58	Inhibits Gli2 nuclear translocation and PTHrP expression in tumor cells	(Vanderburgh et al., 2019); (Vanderburgh et al., 2020)
nHA	Mitochondrial-dependent apoptosis	(Zhang, Zhou, et al., 2019); (Sun, Chen, et al., 2016)
BMS-777607/ASLAN002	Inhibit MSP/RON signaling pathway	(Andrade et al., 2017)
Ga	Decrease RANKL-induced initial expression and auto-amplification of NFATc1	(Strazic-Geljic et al., 2016)
Quetiapine	Inhibiting RANKL-mediated MAPK and NF-κB signaling pathways	(Wang et al., 2015)
PKI-402	Impair the PI3K-AKT-mTOR signaling pathways	(Yuan et al., 2019)
Dasatinib	Inhibit Src activity	(Irelli et al., 2016)
Chinese herbs extractions/plant extractions:		
Licorice isoliquiritigenin (ISL)	Anti RANKL-induced osteoclast generation	(Sun, Zhang, et al., 2019)
Curcumin	Suppress gene products regulated by NF-κB	(Wang, Guo, et al., 2018); (Yang et al., 2020); (An et al., 2018)
Quercetin	Anti-oxidant	(Forte et al., 2017); (Zhang et al., 2017); (Ge et al., 2020)
Plumbagin (PL)	Abrogated RANKL-induced NF-κB and MAPK pathways by blocking RANK association with TRAF6 in osteoclastogenesis	(Qiao et al., 2017); (Li et al., 2012)
Artemisinin, Dihydroartemisinin	Reduced expression of genes involved in osteoclast formation, suppressed osteoclast differentiation, F-actin ring formation and bone resorption through suppressing AKT/SRC pathways	(Ma et al., 2018); (Li et al., 2019); (Feng et al., 2016)
Raddeanin A	Inhibit SRC/AKT signaling pathway;	(Wang, Mo, et al., 2018)
Platycodin D	Blocked RANKL-induced osteoclast formation by inhibiting the expression and nuclear translocation of NFATc1 and c-Fos in BMMs; inhibited the growth of MDA-MB-231 cells	(Lee et al., 2015)
wedelolactone	Inhibit Akt/mammalian target of the rapamycin signaling pathway (mTOR); suppress NF-κB/c-fos/NFATc1 pathway	(Hsieh et al., 2015); (Liu, Hong, et al., 2016)
Thymoquinone	Suppress NF-KB and MAPK signaling	(Thummuri et al., 2015)
Brucine	Suppress Jagged1/Notch1 Signaling Pathways; Increase both the OPG mRNA/RANKL mRNA expression ratio and the OPG protein/RANKL protein ratio	(Hu et al., 2017); (Wu et al., 2017)
benzyl isothiocyanate	Down-regulate RANKL and runt-related transcription factor 2 (RUNX2)	(Pore et al., 2018)
Camellia sinensis	Anti-oxidant	(Luo et al., 2015)
Sinomenine	Reducing IL-8/CXCR1 and c-Fos/NFATc1 signaling	(Zhang, Zou, et al., 2019)

Some traditional Chinese herb extractions were studied for their ability to inhibit the activation of osteoclast induced by cancer cells and obtained optimistic results, as showed in Table 1. Curcumin, extraction from *Curcuma longa* plant, was able to anti-inflammatory and anti-cancer cells through NOTCH-1 signaling and suppress NF-κB relative gene expression. Artemisinin and its derivants, famous for curing malaria, were also researched about their inhibiting osteolysis and cancer ability. Dihydroartemisinin suppressed osteoclasts differentiation, F-actin ring formation, and bone resorption through AKT/SRC pathways. It also inhibited breast tumor-induced osteolysis by inhibiting the proliferation, migration and invasion of MDA-MB-231 cells via modulating AKT signaling pathway. Wedelolactone, a natural product from *Ecliptae herba*, was reported to be capable of suppressing breast cancer-induced osteoclastogenesis and enhancing osteoblastogenesis. And some extractions were revealed effective in suppressing osteoclasts formation and maturity without a clear mechanism yet. In spite of the satisfying experimental outcome, drugs’ low bioavailability, poor solubility, and short half-life limit their application (Wang, Sun, et al., 2016).

In addition to the routine anticancer chemotherapeutic drug, photothermal agents (PTA) have aroused great interest of scientists because of their good photothermal conversion ability which is fundamental for the physical therapy of cancer.

### 3.2. Photothermal materials

**Figure 3.**
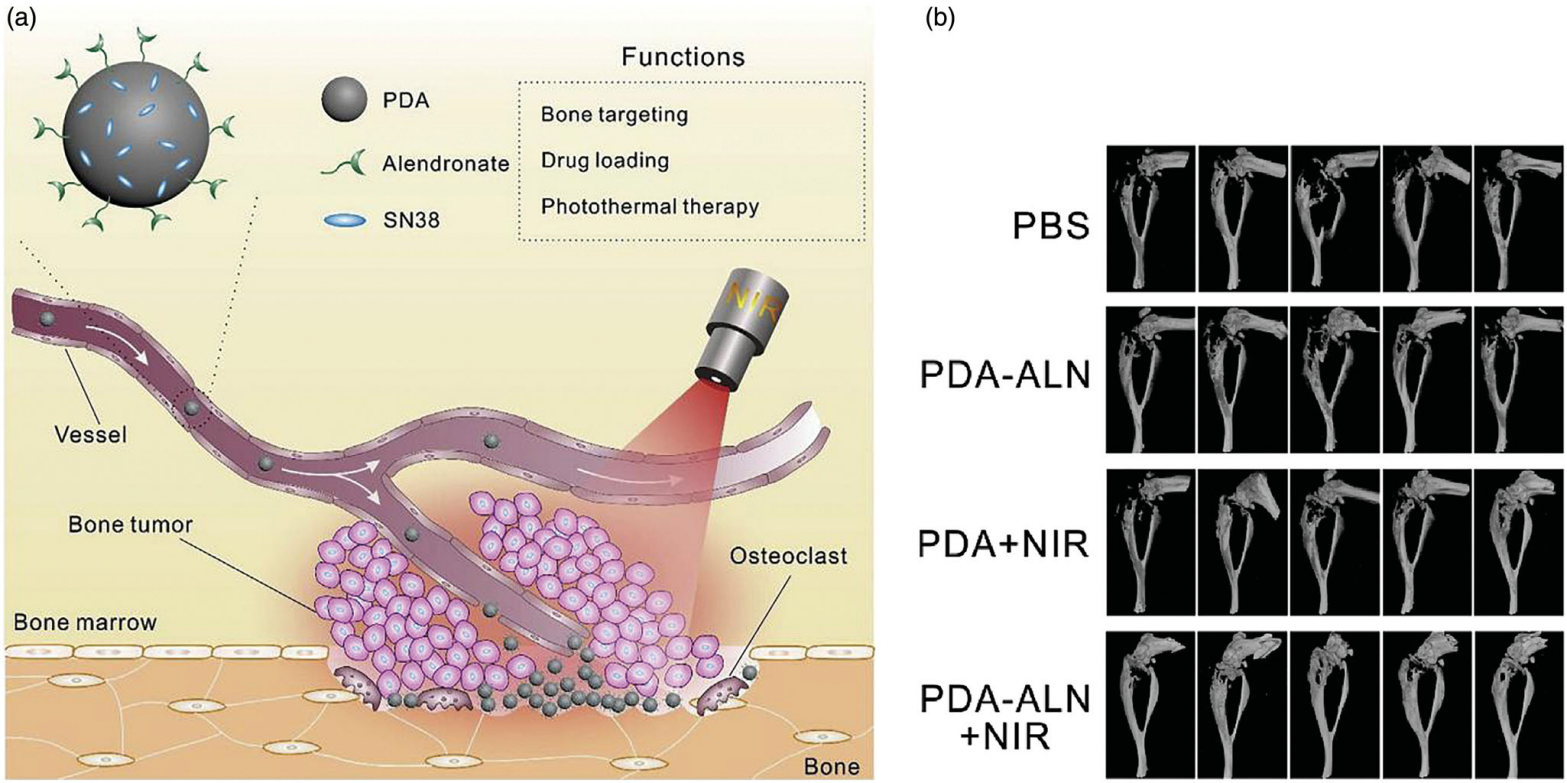
(a) Illustration of bone-targeted NPs with chemo-photothermal treatment. (b) 3D micro-CT reconstruction image of the tumor-bearing tibias after several treatment (Wang, Huang, et al., 2018).

NIR, which is absorbed in minimal amounts by water and biomolecules, can penetrate tissue deeply (Chen, Ning, et al., 2019). First NIR window light (650–950 nm) is often utilized in photothermal therapy (PTT). Exposed to NIR radiation, PTA can generate heat through plasmon resonance or jump energy to rapidly increase the local temperature to ablate cancer cells. Cancer cells are more sensitive to heat than surrounding normal cells and more likely to be damaged with the cells membrane, intracellular protein, or RNA/DNA repair being broken (Abadeer & Murphy, 2016). Regional hyperthermy can kill cells and increase the cells’ sensitivity to anticancer drugs (Sun, Ge, et al., 2019). Frequently-used PTA include inorganic materials (noble metal materials, transition-metal chalcogenide materials, carbon-based nanomaterials etc.) and organic materials (polypyrrole and polydopamine, cyanine dyes, porphyrin, etc.). Gold NPs, Pt NPs, Fe_3_O_4_ NPs, and carbon-based nanomaterials have been explored as PTA because of their effective photothermal conversion (Liu, Bhattarai, et al., 2019; Zhao et al., 2020). Polydopamine (PDA) is formed by mussel-inspired polymerization dopamine. PDA possesses not only versatile adhesion property (through covalent binding or non-covalent binding of catecholamine groups coordination or chelation) which can be utilized to modify the surface of various materials and bind drugs, high chemical activity, excellent biocompatibility, but also strong photothermal property. The biodegradable monodispersed bioactive glass (BGN) was modified by PDA and linked DOX to these NPs for enhancing colon cancer photothermo-chemotherapy and bone repair. NIR-excited PTT of BGN@PDA could ablate tumors. And the relative tumor volume was reduced to 0.85 in BGN@PDA-DOX group, 0.50 in BGN@PDA + NIR group, and 0.01 in BGN@PDA-DOX + NIR group after 12 d of treatment. *In vivo* experiment and quantitative analysis revealed that BGN@PDA and BGN could enhance new bone formation, and NIR did not reduce the bone regeneration efficacy of BGN@PDA (Xue et al., 2020). In another study, the researcher fabricated a kind of ALN-conjugated PDA NPs (PDA-ALN), which exhibited excellent photothermal effect and high affinity to HA. PDA-ALN loaded anticancer drug 7-ethyl-10-hydroxycamptothecin (SN38) via π–π stacking and the release of SN38 could be triggered by NIR. Figure 3(a) showed the mechanism of these multifunctional NPs. The combination of PTT (43 °C) and chemotherapy in this smart and targeted delivery system suppressed the breast tumor’s growth in bone and lessened the osteolytic destruction (Figure 3(b); Wang, Huang, et al., 2018).

Besides, some interesting photothermal materials with smart decoration were synthesized and applied in targeted delivery systems. Na_x_WO_3_ NPs (about 150 nm oxygen vacancy-rich tungsten bronze NPs) were synthesized for PTT. Na_x_WO_3_ NPs had desirable PTT effects that the temperature increased from 25.8 °C to 41.8 °C in 5 min under the irradiation of 980 nm laser. The cytotoxicity of the particles against breast cancer cells appeared in dose-dependence *in vitro*. In breast cancer models, Na_x_WO_3_ + PTT group had the most effective inhibition of tumor and bone destruction (Jie et al., 2019). Except for organic photothermal materials, noble metal and inorganic materials both have nonnegligible cytotoxicity, low biodegradability and low biocompatibility. Effective PTT needs the desired accumulation of PTA in the irradiated area. Thus, it is necessary to apply targeted or local drug delivery systems to carry these PTA to narrow their distribution and modify them to improve biocompatibility.

## 4. Drug distribution optimization

### 4.1. Targeted delivery

As just mentioned, anticancer treatment is the foundation of anti-metastatic osteolysis but inadequate without specific distribution in the osteolytic part or synergy with anti bone resorption drugs. Optimized targeted delivery systems usually rely on NPs which could target metastatic bone and carry anticancer or anti-osteolysis drugs efficiently via targeting ligands. Table 2 listed the collected targeted drug delivery systems. Through systemic administration methods like intravenous injection, targeted delivery systems narrow the drug’s distribution to the bone, tumor, or osteolytic site. The ligands like BP, tetracycline, chelating compounds, salivary proteins, Alizarin Red S, and oligopeptides (oligo-aspartic acid) have an affinity to bone (de Miguel et al., 2014; Chang et al., 2016; Ryu et al., 2016; Stapleton et al., 2017). In the affinity assay for HA *in vitro*, 77.5% of alendronate (ALN, Ald, or Alen) bound to HA powder (Ryu et al., 2016). Zol exhibited 20-fold increased targeting of bone metastatic lesions than other major tissues (Hatami et al., 2019). BP was used mostly to modify the designed NPs which encapsulated anticancer drugs. With BP, NPs can target bone and release anticancer drugs to inhibit cancer cells and suppress osteoclasts. Loaded drugs can be chemically conjugated to the nanocarriers or physically encapsulated or adsorbed and released by diffusion, erosion of carriers or the break of chemical bonds (Zeng et al., 2019). The physicochemical property of these targeted nanocarriers makes a difference in their application (Danaei et al., 2018). The size of nanocarriers cannot exceed 400 nm in order to escape from macrophage and renal clearance and are preferably less than 200 nm (Perez-Herrero & Fernandez-Medarde, 2015). Smaller NPs can help them through the openings of the bone’s sinusoidal capillaries (10–70 nm). Small neutral NPs (about 150 nm) were also 7-fold more effective in localizing in bone marrow than large NPs (about 320 nm; Adjei et al., 2016). Nanocarriers between 70 and 100 nm were often used to target bone for their extended blood circulating time (Adjei et al., 2016). The nanocarriers with hydrophilic, neutral, or slightly anionic surfaces are more prone to localize in the bone marrow and escape from the macrophage attack by avoiding plasma proteins. A neutral surface charge is achieved by modulating the surface-associated emulsifier composition. Hydrophilic surface can be obtained by decoration with hydrophilic polymers like poly(ethylene glycol) (PEG) or amphiphilic polymers like synthetic copolymers of polyethylene oxide (hydrophilic block) and propylene oxide (hydrophobic block) (Perez-Herrero & Fernandez-Medarde, 2015; Suk et al., 2016). Targeted drug delivery systems have been developed based on polymer NPs, micelles, liposomes, Silica NPs, inorganic compounds (HA nanocrystal, calcium phosphates NPs, etc.), or organometallic compounds. Polymer NPs were used most frequently in drug release systems for their good biocompatibility and biodegradability and drugs were released through NPs erosion. Related synthetic polymers include polyglutamic acid, polyglycolic acid (PGA), PEG, polycaprolactone (PCL), polylactic acid (PLA), poly aspartate (PAA), poly(D, L-lactide-co-glycolic) acid (PLGA), and N-(2-hydroxypropyl)-methacrylamide copolymer (HPMA) Ulbrich et al., 2016). Natural polymers include chitosan (CS), alginate, dextran, heparin, albumin, gelatin, and collagen (George et al., 2019).

**Table 2.** Designed targeted drug delivery systems based on nanomaterials or nanotechnology and their release characteristics.

Classification	Loaded drugs	Ligand	Target	Trigger	PTT	Reference
MSNs	DOX, ZOL	ZOL	Bone	pH		(Sun, Han, et al., 2016)
	PL, ZOL	ZOL	Bone	pH		(Qiao et al., 2017)
	ZOL, Au	ZOL	Bone		Yes	(Sun, Ge, et al., 2019)
Calcium phosphate NPs	MTX, ALN	ALN	Bone			(Chu et al., 2017)
Liposome	DOX	Asp8, folate	FR, bone			(Ke et al., 2017)
	ALN, DOX	ALN, hyaluronic acid	CD44, bone	Redox		(Feng et al., 2019)
nMOFs	ZOL	Folate	FR	pH		(Au et al., 2016)
Bioactive glass NPs	DOX			pH, NIR	Yes	(Xue et al., 2020)
Micelles	ALN, curcumin	ALN, Oligosaccharide of hyaluronan	CD44, bone	Redox		(Wang, Guo, et al., 2018)
	BTZ, ALN	ALN	Bone	pH		(Zhu et al., 2018)
	Docetaxel	Quinolone nonpeptide	IntegrinaVβ3			(Ross et al., 2017)
	DTX, ALN	ALN	Bone			(Liu, Romanova, et al., 2019)
QD	ALN, DOX	ALN	Bone	pH		(Li et al., 2017)
Tungsten bronze nanoparticles					Yes	(Jie et al., 2019)
Polymeric NP	DOX, ALN	ALN	Bone			(Rudnick-Glick et al., 2016)
	Cabazitaxel, ALN	ALN	Bone			(Gdowski et al., 2017)
	ZOL	ZOL	Bone			(Hatami et al., 2019)
	GANT58, ALN	ALN	Bone			(Vanderburgh et al., 2020)
	DOX, ALN	ALN, hyaluronic acid	Bone, CD44	pH, redox		(Zhao et al., 2017)
	GANT58			ROS		(Vanderburgh et al., 2019)
	PTX, ALN	ALN, folate	Bone, FR			(Chen et al., 2020)
	SN38, ALN	ALN	Bone	pH, NIR	Yes	(Wang, Huang, et al., 2018)
Dendrimer	Pt NPs	Carboxyl terminals	Bone		Yes	(Yan et al., 2019)
	BTZ	RGD	IntegrinaVβ3	pH		(Wang, Cai, et al., 2018)
	DTX, ALN	ALN, hyaluronic acid	Bone, CD44	pH, redox		(Bai et al., 2019)

#### 4.1.1. Mono target delivery

Most targeted NPs had only one kind of targeting ligands decorated to combine with one specific molecular structure in the osteolytic microenvironment. PLGA and cabazitaxel were used as the core with amino-BP surface conjugation to make NPs. Bone-targeted NPs showed an 8-fold affinity to the bone in 72 h compared with non-targeted NPs. After one-month treatment of bone metastatic models, only the targeted NPs treatment group had a significant reduction in limb weight and pain response. And targeted NPs provided all treated mice protection from bone lesions while 33% of the mice with nontargeted NPs treatment had bone lesions (Gdowski et al., 2017). Similarly, polymeric NPs or micelles encapsulating anticancer drugs, functionalized with ZA or ALN for homing bone, were designed in many other studies and demonstrated much higher efficiency and accumulation in bone than free drugs (Rudnick-Glick et al., 2016; Hatami et al., 2019; Liu, Romanova, et al., 2019). A novel preparation of polymeric NPs with high affinity to bone was completed by Yuya Hirano et al. (Hirano & Iwasaki, 2017). Two-step synthesis of cholesteryl-functionalized poly(ethylene sodium phosphate) (Ch-PEPn·Na) was performed via ring-opening polymerization of cyclic phosphoesters and the demethylation. Synthesized PEPn·Na NPs adsorbed onto HA with the help of poly(ethylene sodium phosphate) which is an analog of polyphosphoesters and has a polyphosphodiester backbone. Scanning electron microscopy images demonstrated PEPn·Na NPs had a higher affinity to the bone than PEG NPs. In addition to polymeric NPs, calcium phosphate NPs and multi-walled carbon nanotubes were also utilized as BP-modified drug delivery systems to target bone (Chu et al., 2017; Dlamini et al., 2019). It was reported that carboxyl groups on the dendrimer surface endowed dendrimer with the intrinsic bone-binding affinity (Yan et al., 2019). The dendrimer loaded with Pt NPs (DEPt-COOH) showed a high affinity to HA and bone fragments *in vitro* and was able to selectively recognize the osteolytic bone lesions *in vivo* (Yan et al., 2019). Polyphosphate (polyP) was also used in a bone-targeted system. Ca-polyP nano/microparticles complexed with ZA maintained the morphogenesis and mineralization-inducing activity of polyP and anti-osteolysis effect of BP (Müller et al., 2018). Optimized PTT systems usually depended on targeted NPs or locally implanted materials to increase PTT efficiency and reduce the toxicity to other organs because PTA are hard to degrade. For example, Gold nanorods were enclosed inside targeted mesoporous silica NPs (MSNs NPs) for PTT and obtained obvious treatment effects (Sun, Ge, et al., 2019).

#### 4.1.2. Dual target delivery

**Figure 4.**
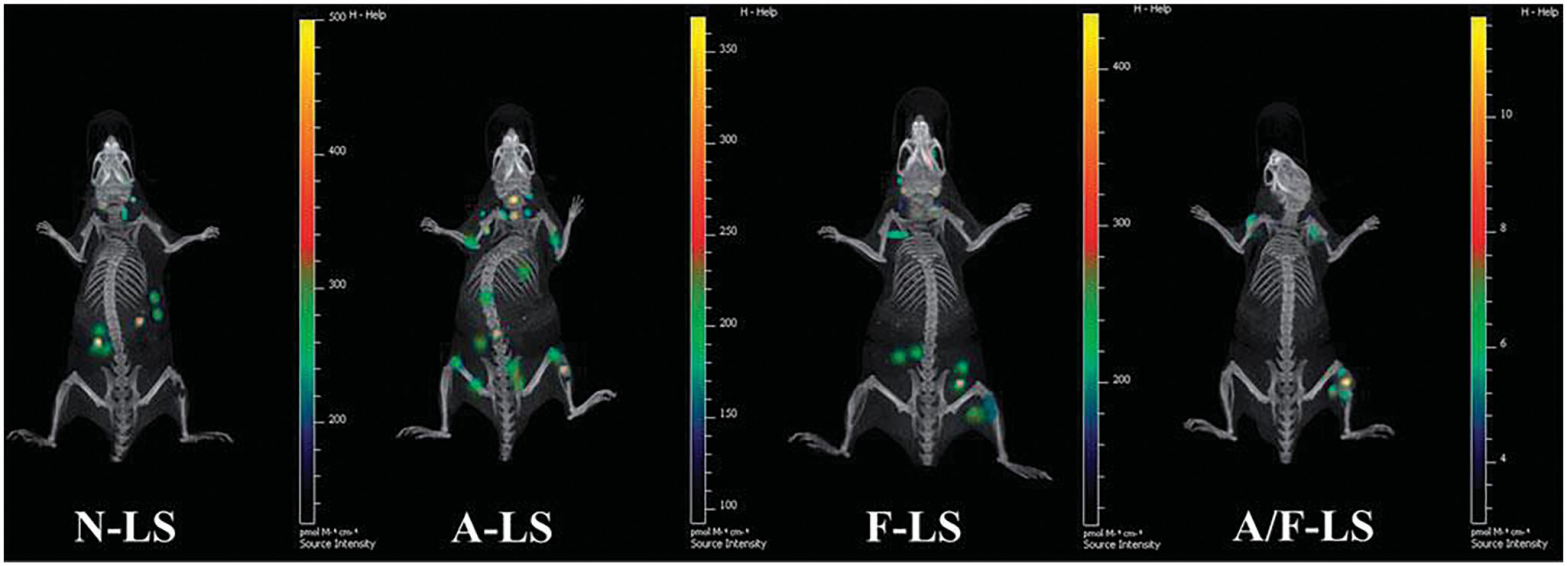
Biodistribution of various liposomes containing Cy5.5 in mice bearing MDA-MB-231 tumors determined by an IVIS Spectrum-CT (Ke et al., 2017).

BP modified drug delivery systems targeted both healthy and destructed bones no matter whether cancer metastasis happened. Adding the targets of delivery systems is an effective way to improve the accuracy of targeting. For instance, designed NPs may contain two types of target ligands that target bone and tumor cells respectively. Dual-targeted or even multi-targeted drug delivery systems rely on the discovery of cells’ special expression or special molecular structure in the osteolytic microenvironment. It was found that bone metastatic cancer cells overexpress folate receptor (FR), cluster of differentiation 44 (CD44) and integrin αVβ3. Human cells express and depend on FR to uptake exogenous folate. FR is widely expressed in normal and tumor cells but more in quantity and activity in tumor cells. In tumor cells, FR loses polarity and becomes easier for drugs in circulation to approach. Folate is a target ligand for FR and is often used to modify the targeted NPs synergistically with BP to target metastatic bone lesions (Assaraf et al., 2014; Au et al., 2016). Shih-Hong Chen et al. designed an NPs-based dual-targeted delivery system comprising hydrophobic PLGA core and ALN-modified, folic acid-conjugated D-a-tocopheryl polyethylene glycol succinate (TPGS) coat to deliver PTX. PTX-loaded dual-targeted NPs evidently accumulated in bone metastases after intravenous injection and exhibited the highest efficiency of inhibiting 4T1 tumor growth. It retarded bone destruction of the tumor-bearing mice appreciably with significant improvement of the survival rate of treated mice (median survival >50 d, but <35 d in other groups; Chen et al., 2020). In another work, aspartate which possesses affinity to the bone because of the similar HA-binding domain of osteopontin and osteocalcin, and folate were used to conjugate onto the surface of DOX-loaded liposomes. Fluorescent images (Figure 4) demonstrated that dual-targeted liposomes optimized the liposome accumulation in cancer metastatic bone (Ke et al., 2017). CD44 is one kind of transmembrane glycoprotein that binds with extracellular matrix hyaluronic acid to mediate intracellular signaling. It is overexpressed in tumor cells and plays an important role in tumor metastasis and invasion. Hyaluronic acid, as a specific ligand for CD44 receptor, was used in many targeted delivery systems (Dosio et al., 2016; Bai et al., 2019). Kaili Wang et al. fabricated CD44 and bone dual-targeted nano micelles via a series of esterification reactions, with oligosaccharides of hyaluronan targeting CD44 and ALN targeting bone. *In vivo* distribution experience, dual-targeted micelles group had higher distribution to the bone than other groups (Wang, Guo, et al., 2018). Another work about DOX-loaded bone and CD44-dual-targeted delivery system showed that the signals of dual-targeting group peaked at 2 h after injection, quicker than the CD44-single-targeting group (6 h). The median survival time was extended to 67 d from 30 d for the untreated group (Feng et al., 2019). These results showed that dual-targeted delivery systems have a stronger ability to target metastatic bone, further optimized the distribution of drugs. Integrin αVβ3 is expressed rarely in normal cells but highly in tumor cells and neoplastic neovascular endothelial cells. It can activate matrix metalloproteinase 2 (MMP-2) and accelerate tumor cells secreting cell adherence molecule which contributes to tumor metastasis and neoplastic neovascular formation. ITGβ3 (a subunit of integrin αVβ3) is over-expressed in bone metastatic cancer cells compared to cancer cells from other metastatic organs such as liver, lymph nodes (Ross et al., 2017; Gdowski et al., 2019). ITGβ3 is a critical factor that contributes to the ability of cancer cells to specifically home and bind to endothelial cells in bone (Kwakwa & Sterling, 2017). This targeting bone function of integrin was exploited to engineer a programmable-bioinspired NPs (P-BiNP) to target bone and increase uptake in homotypic tumor cells (Gdowski et al., 2019). Researchers employed chemokine factor motif chemokine ligand 12 (CXCL12) to stimulate cancer cells to express more integrin, and purified cancer cell membranes to carry PLGA NPs. *In vivo* bone homing experiment, P-BiNP had an increased affinity to the bone and decreased affinity to other organs compared to the BiNPs (Gdowski et al., 2019). On the other hand, integrin αVβ3 can be a target and recognized by tripeptide Arg − Gly − Asp (RGD) and quinolone nonpeptide specifically (Wang, Cai, et al., 2018). Michael H. Ross et al. used quinolone nonpeptide to decorate phospholipid/polysorbate-80 micelle NPs which loaded DOX and demonstrated a stronger accumulation in bone metastases than nontargeted NPs through immunofluorescent analysis *in vivo* (Ross et al., 2017). Well-design and mature technology are required for linking two kinds of ligands to nanocarriers. And whether dual-targeted systems could have better biodistribution still needs to be experimented.

### 4.2. Local delivery

Through the systemic administration, targeted NPs arrive at the destination with the help of the bloodstream. The degradation and drug release of miss-targeted particles may impact the liver or kidney consequently (Chew & Danti, 2017). However, the implanted drug-loading materials deliver drugs directly and locally in implanted position, escaping the ‘danger’ in the bloodstream without facing the blood marrow barrier. Some studies revealed that bone metastasis models treated with locally administered ZA obtained a statistically significant increase in bone volume/tissue volume compared with treatment with systemic ZA administration (Nooh et al., 2017; Ahangar et al., 2018). Local drug delivery systems can further improve drugs’ curative efficiency. These systems rely on implantable biomaterials and implantation operation, maximize the therapy efficiency of drugs, and minimize the toxicity to other organs (Newman & Benoit, 2016). They can reduce the frequency of drug administration because of a large number of drugs loaded and more sustained release, but run the risk of infection and inflammation after implantation (Sarigol-Calamak & Hascicek, 2018). When the tumors in bone marrow develop and cause bone destruction, the operation is necessary. After tumor resection, bone grafts are always implanted as a substitute for destroyed bone (Chen, Boda, et al., 2018), but not enough to inhibit ongoing osteolysis and residual cancer cell proliferation. Therefore, these implanted materials can be modified as multifunctional local drug delivery systems to prevent further bone resorption in subsequent therapy and as bone grafts for the support of the bone function. The implantable materials for lessening osteolysis mainly included scaffolds and porous composite that need to be surgically implanted and locally injected self-assembly hydrogel. Titanium, polymers, calcium phosphate, CS, collagen, hyaluronic acid, alginate were commonly used to fabricate or modify these implantable materials. Solid-state processing (powder metallurgy, sintering of powders and fibers), liquid state processing (direct foaming and spray foaming), vapor deposition, and electrode position were exploited to fabricate porous materials (Sarigol-Calamak & Hascicek, 2018). 3D-print technology (Do et al., 2015; Wang et al., 2020) and electrospinning (Chen, Boda, et al., 2018) were also employed to fabricate 3D multifunctional scaffolds (Yu et al., 2016). Porosity, biodegradability, swelling, and mechanical property of materials have an effect on local drug delivery manner (Chen, Boda, et al., 2018; Wu et al., 2018).

#### 4.2.1. Implantable scaffold or composite

**Figure 5.**
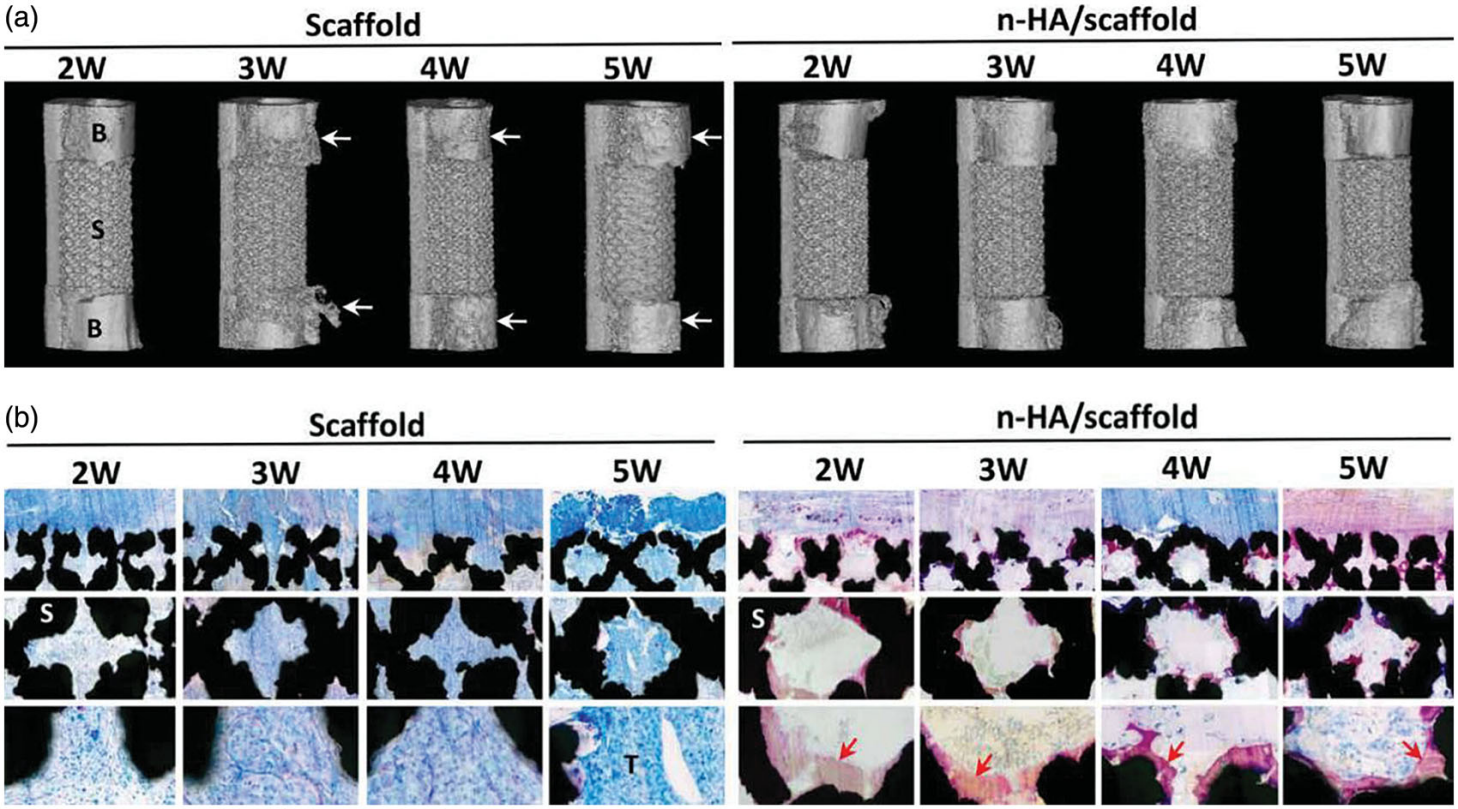
(a) Micro-CT–reconstructed images of the implants and adjacent bone tissue. B: bone; S: scaffold; arrows show adjacent cortical bone resorption by tumor. (b) Histological observation of the implanted scaffolds. S: scaffold; T: tumor; red arrows indicate new bone formation (Zhang, Zhou, et al., 2019).

The most common way to optimize the grafts for treating osteolysis is decorating them with anti-osteolysis drugs. For example, ZOL-loaded bone cement or tricalcium phosphate (TCP) inhibited malignant bone tumors or metastatic bone tumors with the help of sustained ZOL release and acted as a bone substitute at the same time (Koto et al., 2017; Rahmanian et al., 2017). Aliphatic polyesters like PCL, PLA, PLLA were used widely for their good biodegradability, biocompatibility, and permeability (Oledzka et al., 2017). Ewa Oledzka et al. prepared a new multifunctional composite as a promising bone substitute material in which HA porous granules doped with selenite ions (SeO_3_^2−^) and conjugated biodegradable branched copolymer-BP. This composite delivered pamidronate locally to inhibit bone resorption and osteoclast activity. The different molar ratios of copolymers (PLLA/PCL) resulted in different drug release kinetics and degradation speed (Oledzka et al., 2017). In another work, PCL matrix and multilayer polyelectrolyte were combined to fabricate a scaffold in which CaCO_3_ microparticles were covered with multilayer polyelectrolyte and then loaded with dexamethasone (DXM) for sustained local release. As a drug delivery system, this PCL scaffold also promoted osteoblast-like cells (Palamà et al., 2017). 3D-print technology is popular in medicine and bionics. It has been used extensively in fabricating individualistic, multifunctional scaffolds and given the inspiration to design local drug release systems. In the work of Pouyan Ahangar et al., 3D-printed scaffolds which loaded and locally delivered DOX to inhibit cancer cells could also potentially serve as a substitute for destructed bone tissue (Ahangar et al., 2018). Commonly applied for the repair and replacement of bone in the clinic, titanium is often utilized to fabricate 3D bone scaffolds in researches (Wang et al., 2019). Kun Zhang et al. fabricated a 3D-printed multifunctional titanium scaffold with an anti-tumor function via modification of locally-released nHA. After 5 weeks of implantation, *in-situ* tumor volume of the rabbit implanted with nHA/titanium scaffold was 73.8% smaller than other groups. Micro-CT showed nHA/titanium scaffold inhibited osteolytic destruction (Figure 5(a)). New bone formed at the pore walls of the nHA/titanium scaffold (Figure 5(b)) (Zhang, Zhou, et al., 2019). Another optimized implantable titanium scaffold was fabricated with titania nanotube arrays to form nano-engineered 3D titanium wire. TNF-related apoptosis-inducing ligand (TRAIL) was loaded onto titania nanotube arrays (Ti-TNT) to form TRAIL-TNTs for localized cancer treatment (Song et al., 2018). The mechanical property and microstructure of TRAIL-TNTs made it proper to support bone and act as a substitute graft with local treatment after cancer resection. Type I collagen and calcium salt are the main ingredients of bone. Thus, composites containing some similar nature polymers and calcium salt possess good biocompatibility and outstanding ability to induce osteogenesis. Mehdi Rahmanian et al. developed a gelatin/beta-TCP (β-TCP) nanocomposite scaffold loaded with ZA for bone defect regeneration and local drug release after cancer resection. Porous structure (50–200 μm pore) of gelatin reinforced with β-TCP spherical NPs (around 90 nm diameter) facilitated bone cell proliferation. Local ZA release not only inhibited the residual cancer cells and osteoclasts but also promoted osteoblasts proliferation. Histopathological results showed that the new bone formation was established by more than 75% in the whole area of the defect after 3 and 4 months (Rahmanian et al., 2019). PTT can collaborate with implantable scaffold logically and naturally. Fe_3_O_4_ NPs and hydrated GdPO_4_ nanorods were incorporated in bioactive CS matrix to form multifunctional GdPO_4_/CS/Fe_3_O_4_ scaffolds for tumor PTT and bone tissue regeneration. The Fe_3_O_4_ NPs improved NIR absorption capacity and photothermal conversion efficiency. After 10 min of NIR laser irradiation, the temperature of GdPO_4_/CS/Fe_3_O_4_ scaffolds arrived at 47.7 °C and the local temperature around the GdPO_4_/CS/Fe_3_O_4_ scaffolds arrived up to 45.4 °C within 20 s. The tumor diameter in GdPO_4_/CS/Fe_3_O_4_ treatment group decreased with the therapy time extension. Hydrated GdPO_4_ nanorods served as a novel bioactive component for enhancing angiogenesis and osteogenesis. The scaffolds also promoted the osteogenic related gene expression and bone regeneration (Zhao et al., 2020). Multifunctional scaffolds have certain mechanical properties and space structures and these make them only apply to the severe metastatic bone defect area which need surgery.

#### 4.2.2. Injectable hydrogel

Injectable drug delivery systems for treating osteolysis mainly include particle and hydrogel. Hydrogel materials incorporated with drugs and NPs have been explored as local delivery systems. The designed injectable hydrogel can solidify with certain environmental conditions. They were supposed to solidify at about 37 °C through molecular self-assembly. It is conceivable that injectable hydrogel materials have much weaker mechanical properties than implanted scaffolds and this makes hydrogel inferior to scaffolds as bone substitutes. These materials achieved local delivery through simple local injection, not implant surgery. They were also employed for cancer treatment by many researchers (Norouzi et al., 2016). For treating osteolysis, pamidronate (Pami)-derivative and Alen-derivative hydrogelators Pami-D and Alen-D were used to form supramolecular hydrogels under acidic conditions. Pami-D and Alen-D inhibited the proliferation and osteoclastogenesis of bone marrow-derived macrophages in a dose-dependent manner *in vitro* (Tang et al., 2016). This design enriched the application of BP in the realm of the hydrogel. Injectable hydrogel materials, possessing the property of sustained release, are good drug carriers for treating some diseases with low requirement of the mechanical property.

## 5. Drug release optimization

### 5.1. Responsive release

**Figure 6.**
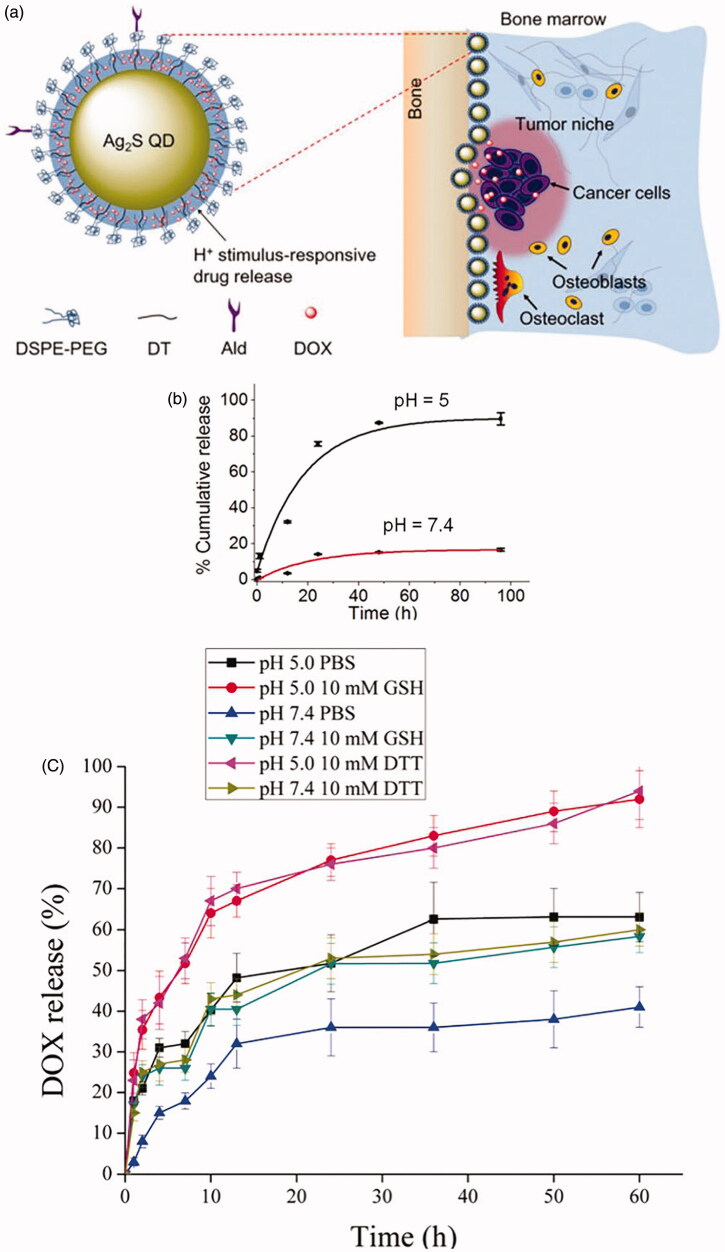
(a) Schematic representation of Ald/DOX@Ag_2_S for bone tumor therapy; (b) Release kinetics of DOX from Ald/DOX@Ag_2_S over time in PBS at pH 5 and 7.4, respectively (Li et al., 2017). (c) Release kinetics of DOX from DOX@ALN-(HA-PASP)CL, GSH (glutathione), DTT (dithiothreitol) (Zhao et al., 2017).

Stimulus-responsive drug delivery systems based on nanocarriers can be triggered by pH, enzymatic catalysis, magnetic, thermal, ultrasonic, electric, and irradiation stimuli (Liu, Yang, et al., 2016; Saravanakumar et al., 2017; Jain et al., 2018). Smart delivery systems for treating osteolysis can be activated by the osteolytic microenvironment such as low pH, high concentration of calcium, and active redox reaction to release drugs responsively (Karimi et al., 2016; Tan et al., 2016; Bai et al., 2019). Designed pH-sensitive delivery systems release drugs through protonation or deprotonation of groups (carboxylic acid group, amino group) or break of chemical bonds (hydrazone, acetal, cis-Aconityl, β-thiopropionate, etc.) in an acidic environment. Aryl boronate group, as an acid–labile linker, was employed to construct pH-sensitive delivery systems. Mingming Wang et al. made polyamidoamine (PAMAM) dendrimer with catechol and PEG ligands modification (Wang, Cai, et al., 2018). Boronate-catechol linkage was pH-responsive and linked bortezomib (BTZ) to dendrimer for responsive BTZ delivery to low pH. BTZ was released from the polymer less than 5% at pH 7.4 at 12 h but nearly 57% at pH 5.0 at 6 h. PH 5.0 is usually known as the lysosomal pH (pH 4.5) (Wang, Cai, et al., 2018). A similar design was made by Jianhua Zhu et al. (Zhu et al., 2018). In a study, Zol-anchored MSNs (DOX@MSNs − ZOL) loaded DOX and had obvious pH-sensitive DOX release behavior because MSNs − ZOL did not have enough hydroxyl radicals to create hydrogen bond with the amino group of DOX. DOX@MSNs − ZOL entered cells through an ATP-dependent pathway and then localized in the lysosome to achieve effective intracellular DOX release (Chen, Boda, et al., 2018). Chunyan Li et al. fabricated a Ag_2_S QD-based nanosystem (Ald/DOX@Ag_2_S) with surface-anchored ALN and encapsulated DOX (Figure 6(a)). These NPs were deposited in bone tissues and then DOX was released in the tumor site triggered by the acidic tumor microenvironment. The drug release kinetics was exhibited in Figure 6(b). Ald/DOX@Ag_2_S caused a time and dose-dependent increase in caspase-3 activity (apoptosis assay) and inhibited the osteoclastic differentiation of RAW264.7 cells stimulated by RANKL (Li et al., 2017). Likewise, redox acted as a trigger in some targeted delivery systems because of the active redox at the osteolytic site. Glutathione (GSH) is an antioxidant and its concentration is 7–10 times higher in tumor cells than normal cells (1–10 mmol L^−1^) (Wu et al., 2004; Aluri et al., 2009; Wang et al., 2014). Disulfide bonds can be broken by GSH so that it was utilized by many scientists to fabricate micelles or polymeric NPs in response to the active redox. J.P. Vanderburgh et al. developed aqueous and intravenously injectable micellar NPs to encapsulate GANT58 via a combination of anionic and reversible addition-fragmentation chain transfer (RAFT) polymerization. Poly[(oligo ethylene glycol)_9_ methyl ether acrylate]_17_ formed the hydrophilic NPs surface while polymer poly(propylene sulfide) formed the hydrophobic core that sequestered GANT58. In response to reactive oxygen species (ROS), poly(propylene sulfide) became hydrophilic and degraded to initiate drug release. In an intratibia breast cancer bone metastasis model, these NPs decreased bone lesion area and lesion number by 49% and 38% respectively, and increased trabecular bone volume (Vanderburgh et al., 2019). Stimulus-release systems also can be triggered by two or more stimuli. Metal-organic frameworks (MOFs), mainly in a cubic shape, with an average size <100 nm in diameter, were utilized to construct a multi-stimuli ‘gated scaffold.’ The frameworks based on zirconium, remained tightly and controlled release at the normal Ca^2+^ concentration, pH, and temperature, but released drugs around/in bone tumor cells with increasing Ca^2+^ concentration, decreasing pH, and/or by using thermal therapy. During this process, carboxylatopillar[5]arene played a vital role in regulating the binding between the rings-structure and the stalk-structure (Tan et al., 2016). Multi-stimuli responsive systems are more sensitive to the osteolytic microenvironment and deliver the drug more rapidly but less specifically. It may be optimized through integration with implantable materials or targeted NPs. Another NPs, DOX@ALN-(HA-PASP)CL, were prepared with pH sensitivity and redox sensitivity. During the synthesis of these NPs, ALN and HA were linked by a hydrazone bond which was responsive to the acidic microenvironment. HA and PASP [poly(aspartic acid)] were cross-linked by a disulfide bond to increase the stability of the NPs. Release kinetics of DOX from DOX@ALN-(HA-PASP)CL was showed in Figure 6(c). Tumor and bone resorption was inhibited obviously in the research (Zhao et al., 2017). The responsive delivery embodied the prevention theory. But if osteolysis already happened, stimulus-release aimed to reduce the drug release of miss-targeted NPs to further reduce the side effects and accelerate the drug release in the osteolytic microenvironment.

### 5.2. Sustained release

**Figure 7.**
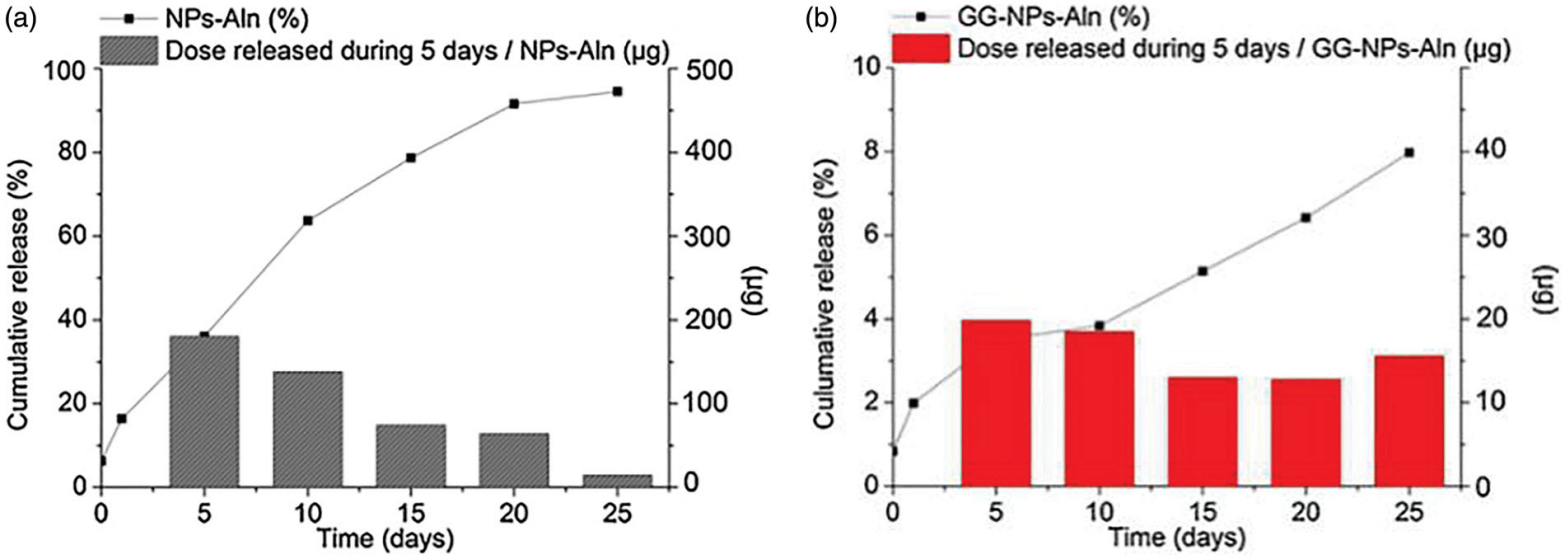
ALN release curves from drug-loaded NPs (a) and gellan gum based injectable system (b). Cumulative curves (black squares and lines) as well as doses released from 1 ml of NPs-ALN suspension (1% w/v) or 1 ml of GG–NPs-ALN system during 5 d intervals (boxes) are shown (Posadowska et al., 2015).

In local delivery systems based on implanted materials, drug loading can be obtained by the following methods: (a). mixing drugs with materials directly before fabrication; (b). entrapping drugs into basic drug carriers (e.g. NPs) and adding this system to implantable materials; (c). immersing scaffold/hydrogel/composite into drugs solution; (d). anchoring drug on the materials (physical adsorption, chemical conjunction); (e). coating the scaffold in a polymer or composite solution (Sarigol-Calamak & Hascicek, 2018). Drugs can be released from implantable materials in several ways, such as drug diffusion, drug adsorption–desorption, and materials erosion (Chen, Boda, et al., 2018). The early-stage release always appears a burst release through diffusion followed by a constant release through materials erosion or further diffusion (Zeng et al., 2019). Biodegradable materials are degraded by hydrolysis and enzymolysis (Song et al., 2018). When NPs serve as drug carriers and then are incorporated in implantable materials or modify the materials, special properties of NPs can endow the implantable materials with new merits, such as stimulus-release ability, photothermal property, and more sustained release ability. GG-NPs-ALN system, with ALN-loaded PLGA NPs carried in the matrix of gellan gum, was injectable and delivered ALN intra-bone in a constant manner. The ALN release graphs of NPs-ALN and GG-NPs-ALN are shown in Figure 7. It was obvious that this local release system released drugs more slowly and constantly. GG-NPs-ALN was cytocompatible with osteoblast-like cells but inhibited RANKL mediated osteoclastic differentiation of RAW 264.7 cells significantly *in vitro* (Posadowska et al., 2015). Liposomes were often incorporated in hydrogel materials (Ding et al., 2016; Wu et al., 2018). Wei Wu et al. designed a local release system (GEM-Lip@Gel) based on hydrogel, in which liposomes blended with methacryloyl (GelMA). Gemcitabine (GEM) was loaded into liposomes for local release to kill osteosarcoma cells. GEM-Lip@Gel (with liposome) had a more constant level than GEM-GelMA (without liposome) in drug release assay and showed the strongest ability to inhibit cancer cells than the control *in vitro* and *in vivo* (Wu et al., 2018). Table 3 outlined some local delivery systems of lessening bone destruction and their drug-release characteristics. A sustained release can be realized easily by biodegradable materials like hydrogels. Biodegradable materials release drugs sustainedly through erosion and it is controllable through regulating the matrix ingredient ratio, crosslinking method, or utilizing NPs which encapsulate drugs.

**Table 3.** Designed local release systems based on nanomaterials or nanotechnology and the released characteristics.

Classification	Matrix	Fabrication methods	Loaded drug	Osteogenesis	PTT	Trigger	50% cumulative release time	Reference
Scaffold composite	Titanium	3D print; slurry foaming method	HA NPs	Yes			>7 d	(Zhang, Zhou, et al., 2019)
	Thermoplastic polyurethane (TPU) copolymer with polyvinyl alcohol	3D print	DOX				∼1 d	(Ahangar et al., 2018)
	PCL	Solvent casting and particulate leaching technique	Dexamethasone	Yes			>192 h	(Palamà et al., 2017)
	Titania	Electrochemical method	TRAIL				>4 d	(Kaur et al., 2016)
	Hydroxyapatite granules and branched copolymer-pamidronate	Copolymerization	Pamidronate				Bis-MPA-PLLA90-PCL38 > 20h;bis-MPA -PLLA64-PCL64,bis-MPA -PLLA38-PCL90 > 120h	(Oledzka et al., 2017)
	Gelatin/(β-TCP) nanocomposite	Cross-linking and self-assemble	ZOL	yes				(Rahmanian et al., 2017)
	Hydroxyapatite (HA) and polymethyl methacrylate (PMMA) bone cement	Polymerization	ZOL	Yes				(Koto et al., 2017)
	CS	Freeze-drying		Yes	Yes			(Zhao et al., 2020)
Hydrogel	Pluronic	Cold method	Bleomycin A6				∼8 h	(Ding et al., 2016)
	Gelatin methacryloyl	*In-situ* photocrosslinkable	Gemcitabine				<∼12 h	(Wu et al., 2018)
	Gellan gum	Cross-linking and reassemble	ALN				>25 d	(Posadowska et al., 2015)
	Pami-D; Alen-D	Self-assemble	Pami-D; Alen-D					(Tang et al., 2016)

## 6. The prospect

### 6.1. Multifunctional nanomaterials

The optimization in this review does not merely focus on drug distribution and release manner but also emphasizes the design of multifunctional materials. Utilizing nanotechnology to realize inhibiting cancer cells and osteoclasts simultaneously is a great optimization and improvable. When metastatic bone destruction occurs, it is not enough to anti-cancer and anti bone resorption, promoting bone regeneration is also important to improve the quality of life. In tissue engineering and regenerative medicine, many implantable biomaterials have been designed with good biocompatibility and osteoconductive ability to promote bone tissue regeneration (Sarigol-Calamak & Hascicek, 2018; Martin & Bettencourt, 2018). Calcium phosphate (Lee et al., 2016), CS (Kozusko et al., 2018), collagen (Cruz-Neves et al., 2017), hyaluronic acid, nHA (Cruz-Neves et al., 2017), polymers (Olthof et al., 2018; Sarigol-Calamak & Hascicek, 2018), metal and some composites (Newman & Benoit, 2016) were often used to fabricate bionic scaffolds with osteogenesis drugs, factors or even cells carried on, like bone morphogenetic protein-2 (BMP-2) (Holzapfel et al., 2016; Lee et al., 2016; Martin & Bettencourt, 2018; Olthof et al., 2018). These implanted bone tissue engineering or bone regeneration materials can be explored as multifunctional materials to carry anti-osteolysis drugs or anti-osteolysis drug delivery systems. Multifunctional materials also are characterized by multi drugs and multi delivery systems. The integration of different drugs and different systems (target delivery systems, PTT systems, local delivery systems) can be realized by tactfully designed nanomaterials and novel fabrication nanotechnology. During the treatment of osteolysis, reducing bone pain is another therapy goal. Designed materials should also be tested for their bone pain alleviating ability, such as mechanical allodynia and thermal hyperalgesia test on cancer metastasis model (De Felice et al., 2016; Wei et al., 2017; Zhou, Chen, et al., 2017).

### 6.2. Gene therapy

Gene therapy aims at regulating cells’ apoptosis and function at the level of genes to treat disease. Most cancer-related gene therapy introduced exogenous DNA segments or delivered siRNA or miRNA into target cells to regulate DNA expression (Fitzgerald et al., 2016; Wang, Yu, et al., 2016; Lima et al., 2019). Till November 2017, almost 2600 gene therapy clinical trials had been completed and about 85% of these trials used a virus as vectors or naked/plasmid DNA (Ginn et al., 2018). However, The clinic applications are limited because of the drawbacks of nucleic acids. The free nucleic acid in blood could be degraded by various cells (reticuloendothelial system, mononuclear phagocyte system) and biomolecules (nucleases). The size of plasmid DNA could hinder it into cells (Zhou, Liu, et al., 2017). Several non-viral vectors were fabricated to deliver nucleic acid, including cationic polymers like polylysine (PLL), poly(ethyleneimine) (PEI), PAMAM, PEG, CS, cationic lipids (Chen, Yue, et al., 2018), inorganic NPs (gold, silica, iron oxide and quantum dot) (Zhou, Liu, et al., 2017). In many types of research, gene therapy cooperated with PTT to anti-cancer relying on nano delivery systems (Kim et al., 2016; Wang, Yu, et al., 2016; Chu et al., 2019). Knockdown of BMPR1a in breast cancer cells through Adenovirus-mediated RNA interference was reported to suppress the production of RANKL via p38 pathway and inhibit cancer-induced osteoclast genesis (Liu et al., 2018). However, few delivery systems of optimizing gene delivery to stop the ‘vicious circle’ were available. Actually, the aforementioned nanomaterials or nanotechnology can be applied to optimize the relevant DNA/RNA delivery to hinder osteolysis.

Exosomes are a hot topic recently. Exosomes are lipid bilayer vesicles containing miRNA and protein. They are produced and excreted from cells to regulate cells’ action and are also explored as drug carriers (Ha et al., 2016; Bellavia et al., 2018).

In osteolysis, some miRNAs play a very important role in regulating bone destruction. MiR-31, miR-33a, miR-34a, miR-133a, miR-141, miR-155, miR-190, miR-192, miR-219, and miR-223 were reported to be capable of influencing osteoclast differentiation in bone metastasis (Ell et al., 2013; Kagiya, 2015). A hydrogel-embedded, gold-NPs-based delivery vehicle provided efficient local, selective, and sustained release of miR-96/miR-182, markedly suppressed metastasis in a breast cancer mouse model (Gilam et al., 2016). Implantable nanomaterials and targeted NPs may provide new ways for applying miRNA or exosome to cure osteolysis.
